# Statistical Evaluation of HTS Assays for Enzymatic Hydrolysis of β-Keto Esters

**DOI:** 10.1371/journal.pone.0146104

**Published:** 2016-01-05

**Authors:** O. Buß, S. Jager, S. -M. Dold, S. Zimmermann, K. Hamacher, K. Schmitz, J. Rudat

**Affiliations:** 1 Technische Universität Darmstadt, Computational Biology and Simulation, Darmstadt, Germany; 2 Karlsruhe Institute of Technology, Biomolecular Separation Engineering, Karlsruhe, Germany; 3 Karlsruhe Institute of Technology, Technical Biology, Karlsruhe, Germany; 4 Technische Universität Darmstadt, Biological Chemistry, Darmstadt, Germany; University of Graz, AUSTRIA

## Abstract

*β*-keto esters are used as precursors for the synthesis of *β*-amino acids, which are building blocks for some classes of pharmaceuticals. Here we describe the comparison of screening procedures for hydrolases to be used for the hydrolysis of *β*-keto esters, the first step in the preparation of *β*-amino acids. Two of the tested high throughput screening (HTS) assays depend on coupled enzymatic reactions which detect the alcohol released during ester hydrolysis by luminescence or absorption. The third assay detects the pH shift due to acid formation using an indicator dye. To choose the most efficient approach for screening, we assessed these assays with different statistical methods—namely, the classical Z’-factor, standardized mean difference (SSMD), the Kolmogorov-Smirnov-test, and *t*-statistics. This revealed that all three assays are suitable for HTS, the pH assay performing best. Based on our data we discuss the explanatory power of different statistical measures. Finally, we successfully employed the pH assay to identify a very fast hydrolase in an enzyme-substrate screening.

## Introduction

*β*-amino acids are intermediates in the synthesis of a great variety of pharmaceutically important compounds [1, 2]. The objective of this study is to select an HTS assay to screen for one enzyme for a two-step reaction cascade for the synthesis of *β*-amino acids. These occur in a number of biologically active compounds such as paclitaxel, bleomycin and the lipopeptide YM-170320 [3, 4]. Paclitaxel is used as a drug in the treatment of certain types of cancers [5]. As the correct configuration of the stereocenters is essential for biological activity, synthesis strategies with high enantioselectivity are desirable [2].

A variety of synthesis strategies have been established for the production of chiral *β*-amino acids. However, the limitations of these strategies are unfavourable for industrial production [6, 7]. In addition to the chemical approaches, enzymatic strategies have been established to produce chiral *β*-amino acids. Since all enzymatic approaches established in a industrial scale are based on kinetic resolutions, their theoretical yield is limited to 50% at most [8]. Both the enzymatic and chemical synthesis strategies are still a subject of research for the production of *β*-amino acids [9]. In order to achieve higher yields than 50%, *β*-amino acids can also be obtained with high enantiomeric excess by enzymatic conversion of *β*-keto acids using transaminase [9]. However, the substrates of this synthesis strategy, the *β*-keto acids, are not stable and decarboxylate. To avoid this side reaction *β*-keto acids can be generated *in situ* by hydrolysis of a *β*-keto ester catalyzed by a hydrolase. In the synthesis of natural and non-natural substrates hydrolases are a beneficial and commonly used enzyme class [10] and a number of lipases and esterases are commercially available [11]. Many enzymes are well characterized, but often there is no perfect match between the requirements of an efficient catalysis reaction and the properties of the biocatalyst. Besides being able to acquire hydrolases commercially, there is the possibility to test hydrolases from different sources, such as biomolecular libraries containing purified enzymes, microorganisms from the environment or hydrolases variants from directed evolution and from in *silico* gene data basis. The limiting factor is the fast and reliable identification of the best fitting enzyme.

### HTS-assays for hydrolases

While investment in both, experimental time and costs, can increase sample throughput in large screenings with standard analytical hardware [12, 13], this route can quickly become prohibitively expensive. High throughput assays permit simultaneous measurement of samples in 96- to 1536-well plates so that 10^5^to 10^7^samples may be screended per day [14–16]. This way, large biomolecular libraries can be screened for optimal enzymes [16].

A wide variety of assays has been established for the detection of efficent hydrolases *in vivo* or *in vitro* [17, 18]. HTS assays for lipases and esterases have been extensively reviewed [19, 20]. HTS assays may directly detect the reaction products or convert these products for indirect detection. For example a simple indirect HTS-assay detects the pH-shift during a hydrolysis reaction by a pH-indicator molecule [21].

Enzyme activity can be detected by a number of parameters such as microbial growth or changes in spectral properties, temperature or electrochemical potential as well as by luminescence [17]. Chromatography methods are well suited for medium-throughput screenings [13]. The assessment of temperature change due to exothermic reactions has been reported, as a technically sophisticated measure requiring specialized infrared cameras, which are no standard laboratory equipment [22]. In direct HTS assays product formation is monitored by a change in a physical quantity associated with the product.

For this purpose, chromogenic and fluorogenic substrates are frequently applied in these assays [18]. Additional analytical reactions are also commonly used [18]. These mostly synthetic substrates allow for an easy readout by changes in absorbance, fluorescence or by chemiluminescence [12]. However, when a substrate analog with an artificial chromophore or chromogenic group is employed in the the optimization process instead of the substrate of interest this may lead to an enzyme optimized processing the analog but not the substrate of interest (“You get what you screen for” You and Arnold *et al.* 1996) [13, 19, 23, 24].

### Disadvantages of assay based on non-natural fluorophores/chromophores

A variety of hydrolase screening assays is based on non-natural substrates with chromophoric groups. Well-known examples are umbelliferyl-, 4-nitrobenzofurazane- and 4-nitrophenyl-assays [25–27]. The disadvantage of this assays is caused by the difference between the properties of the substrate to the non-natural substrate. The most active enzymes in this kind of screening do not have to be the most active enzyme for the natural substrate. As an example, for screening hydrolases, 4-nitrophenyl esters are popular, because standard photometric plate readers can easily monitor the reaction by detection of the colored product at 348 to 405 nm [13]. However, 4-nitrophenyl esters *per se* are no substrate with industrial pertinence [28, 29]. Furthermore, the absorption of the liberated 4-nitrophenol at 405 nm depends on the pH-value, so that the pH value needs to be controlled. Alternatively, absorbance may be measured at the isosbestic point of 4-nitrophenol at 348 nm [30, 31]. Moreover, 4-nitrophenyl esters are more readily hydrolyzed than esters comprising aliphatic alcohol residues like methanol or ethanol. When 4-nitrophenyl esters are used to in hydrolase screening, the hits may therefore exhibit lower activity towards the actual substrate [19, 32]. In 2008, J. Córdova *et al.* [33] reported enzymatic activity of bovine serum albumin (BSA) showing high hydrolysis rates for 4-nitrophenyl esters at 80–160°C. Under these conditions, a spontaneous hydrolysis of 4-nitrophenyl esters is likely, so that the observed hydrolysis may not be necessarily due to an enzymatic activity of BSA. Another argument against the use of 4-nitrophenyl esters is the potential reaction with nucleophilic amino acid side chains as it was shown for the reaction of 4-nitrophenyl acetate with L-tyrosine esters by B.S. Hartley *et al.* in 1953 and the acetylation of insulin in the reaction with 4-nitrophenyl acetate [34]. Modification of the enzyme by acyl-group transfer may lead to artifacts during screening. Taken together, 4-nitrophenyl esters are are often not suitable as substrate analogues for screening. If possible, alternatives to the fluorophoric and chromophoric non-natural substrates should be used.

### Indirect assays as an alternative approach

If it is impossible to directly detect the conversion of the native substrate, the alternative approach is to further convert the products for indirect detection. A simple approach are colorimetric pH-assays, which can be employed if one of the products is an acid that subsequently deprotonates or a base that lowers the proton concentration in the medium. This change in pH value can be monitored by an indicator dye [35, 36]. Enzymatically coupled systems that transform one of the products yielding a detectable compound are more complex. As more reaction steps and compounds required for quantification more parameters need to be optimized and the readout is more prone to errors. One of the first examples for a convenient indirect assay was the NADH-dependent, coupled enzyme assay for urease by Kaltwasser *et al.*(1966) [37].

For the hydrolysis of *β*-keto esters, we compared three different indirect assays for the activity of hydrolases. One assay relies on the change of the pH-value, the second is based on enzymatic oxidation of the released ethanol to acetic acid and the third, which we expected to be most sensitive, is based on the oxidation of ethanol to ethanal and hydrogen peroxide which is then converted by horseradish peroxidase (HRP) in a luminescence reaction. The first assay is the simplest one, because only a buffer- pH indicator system is needed and many examples are established for enzyme screening with pH-indicators [21, 35, 38, 39]. The second assay is also well-known and established for measurements of alcohol in food [40]. This assay was miniaturized to microtiter plates. The third assay is a modification of a chromogenic alcohol-oxidase(AOX)/peroxidase (HRP) ethanol assay for determination of ethanol in beverages, which normally based on 2,2’-azino-bis(3-ethylbenzthiazoline-6-sulphonic acid (ABTS) as chromogenic substrate [41]. This assay was modified to a luminometric assay by adding luminol instead of a chromogenic substrate. The luminometric measurements should be more sensitive due to photons are released by the detection reaction. For the first time the system was established in a flow system with separated bioreactors by Marschall and Gibson [42]. For the quantification of ethanol, we adapted the luminol-AOX-HRP system to microtiter plates by using design of experiments (DoE). The aim was to optimize the system for endpoint measurements of enzymatic hydrolysis reactions. Therefore, for the first time a luminometric AOX-HRP-system was tested for quantification of ethanol in 96-well plates. In this study all assays were tested for HTS compatibility.

### Statistical evaluation methods

To compare the quality of high-throughput assays the Z’factor is frequently employed [14, 43]. In addition, the assessment of measurement procedures can be based on traditional statistic parameters like signal-to-noise-ratio(S/N), signal to background ratio (S/B), and coefficient of variation (CV) (Table 1). CV is the ratio of mean to variance and often used as quality control for assays [44]. The S/B-ratio is a criterion that indicates whether the level of the signal is sufficiently high above the background. The rule of thumb for a good HTS assay is S/B >3. However, fluctuations in both the signal and the background are not considered. In contrast, the S/N-ratio takes into account the standard deviation of the background. The higher the S/N ratio, the less do background fluctuation influence the desired signal. Both measures indicate whether a sample could be distinguished from the background, however they don’t quantify to what extent the positive and negative controls can be distinguished.

**Table 1:** Overview of statistical measures for evaluation of HTS assays. *σ* = standard deviation; *μ* = mean; *m* = median; pos = data set of positive controls; neg = data set of negative controls; *n* = sample size; *F*_*k*_ = cumulative density function for data set *k*; *max*_*j*_ = maximum distance between two distributions; SSMD = Strictly standardized mean difference; *x*_*i*_ = *ith* value of the (ordered) data set x.

**Measures**	**Definition**
Kolmogorov-Smirnov-test (KS-test)	*KS* = max_*j*_(*F*_1_(*x*_*j*_) − *F*_2_(*x*_*j*_))
*t*-statistic	$t=\frac{\mu_{\text{pos}}-\mu_{\text{neg}}}{\sqrt{\frac{\sigma_{\text{pos}}^{2}}{n_{\text{pos}}}-\frac{\sigma_{\text{neg}}^{2}}{n_{\text{neg}}}}}$
Z’-factor	$Z=1-3\frac{\sigma_{\text{pos}}-\sigma_{\text{neg}}}{\mu_{\text{pos}}-\mu_{\text{neg}}}$
SSMD_*MM*_ (method of moment)	$\beta_{\text{MM}}=\frac{\mu_{\text{pos}}-\mu_{\text{neg}}}{\sqrt{\sigma_{\text{pos}}^{2}-\sigma_{\text{neg}}^{2}}}$
SSMD_*ML*_ (maximum likelihood)	$\beta_{\text{MLE}}=\frac{\mu_{\text{pos}}-\mu_{\text{neg}}}{\sqrt{\sigma_{\text{pos}}^{2}\frac{n_{\text{pos}}-1}{n_{\text{pos}}}+\sigma_{\text{neg}}^{2}\frac{n_{\text{neg}}-1}{n_{\text{neg}}}}}$
SSMD_*R*_ (robust)	*MAD* = 1.4826*m*(|*x*_*i*_ − *m*|)
	$\beta_{R}=\frac{m_{\text{pos}}-m_{\text{neg}}}{\sqrt{MAD_{\text{pos}}^{2}-MAD_{\text{neg}}^{2}}}$

Therefore methods have been explicitly developed to evaluate high-throughput screenings, like Z’-factor and the Strictly Standardized Mean Difference (SSMD) [45].

J.H. Zhang (1999) defined the Z’-factor based on the normal distribution and the 3-sigma rule of thumb [43, 46], which implies that 99.7% of all samples lie within less than three standard deviations distance from the mean. The Z’-factor has become a common metric for HTS quality control as it allows to decide whether an assay is suitable to just distinguish positive samples from negative ones (Z’>0,5) or whether it can distinguish well performing samples from poor ones (Z’>0,8) [14, 43, 47].

The strictly standardized mean difference (SSMD) takes the mean difference of negatives and positives in proportion to the standard deviation. SSMD gained recognition in screening e.g. for antiviral drugs [48]. In 2007, X.D. Zhang derived the SSMD under the condition of independence of both distributions through maximum likelihood estimation (MLE) and method of moment (MM). Finally, for increased robustness against outliers, the median of absolute deviation (SSMD_*R*_) can be used.

In this work, we applied for the first time these different SSMDs for evaluation of a biocatalyst screening assay. SSMD have already been used in RNAi screening and cell-based systems or for quantification of enzyme inhibition [49, 50] but never for assays of enzyme catalysis. We compared these measures to the Z’factor.

We also chose non-parametric methods like the KS-test and *t*-statistic. Non-parametric test statistic differs from parametric statistic in that no explicit distribution is given, but the procedure is solely based on the data without the need for any model. The *t*-statistic describes the difference of two sample distributions measured in standard errors of both means. This is suitable for small sample sizes, especially when the underlying distribution is unknown or not derivable [51].

In contrast to all other methods, the Kolmogorow-Smirnow-Test (KS-test) [52] calculates the maximum distance between two cumulative density functions (CDF). The CDF can be computed for finite number of data points as a cumulative sum of the frequency of occurrence of the (sorted) data. The unique benefit of this kind of calculation is that no previous knowledge about distribution or models is needed. We include the KS test here to contrast it with the other measures that do not rely on any CDF.

## Materials and Methods

All enzymes and chemicals used in the assays were purchased from Sigma-Aldrich (St. Louis, USA) and Carl Roth (Karlsruhe, Germany). Reagents were dissolved in 40 mM potassium phosphate buffer (pH 7.2) unless stated otherwise. For all buffers and solutions deionized water was used.

### Expression and purification of para-nitrobenzyl-esterase 13 (pNB-Est 13)

Expression was performed in *E. coli* BL 21 codonplus using a pET-22b vector system with a pelB-leader sequence for export to the periplasm of *E.coli*. After transformation cells were cultivated at 180 rpm and 30°C in shaking flasks in 400 mL LB-medium. After the OD600 had reached 0.4, expression was induced with isopropyl *β*-D-1-thiogalactopyranoside (IPTG, Carl Roth, final concentration 0.2 mM). The expression proceeded over 15 h at 20°C. The cells were harvested by centrifugation at 4700 rpm for 20 min at 4°C (Heraeus Multifuge X3 FR). Protein purification from the periplasm was carried out by osmotic shock in ddH_2_O, using the protocol by Petersen *et al.* [53]. The supernatant was frozen in liquid nitrogen and lyophilized overnight (Lyophilizer, Beta 1–8 Martin Christ). The product was analyzed by SDS-polyacrylamide-gel electrophoresis (SDS-PAGE, S2 Fig).

### Oxidative luminescence assay

Using the standard conditions of the AOX-HRP-ABTS system (described in the protocol of Sigma-Aldrich [54]) the luminescence signal was very weak for ethanol (2.0 mM) [55, 56]. To gain a much longer and more intensive luminescence reaction a design of experiments with a statistic based optimization program (Modde 10.1 Software (Umetrics, Sweden)) was carried out (S4 Fig and S5 Fig). For measuring luminescence, white 96-well plates (Greiner, Austria) were used. The final reaction volume per well was 200 μL. Reaction mixtures were prepared on a Tecan Freedom Evo 200 liquid handling station (LHS). The LHS is equipped with one eight-port liquid handling arm (LiHa), a standard robotic plate handling arm (RoMa) equipped with a centric gripper, a 96-channel liquid handling arm (MultiChannelArmTM (MCA 96), Tecan) with an eccentric gripper, and an integrated spectrophotometer (Infinite M200 Pro, Tecan). Pipetting precision and accuracy of aqueous solutions was determined by pipetting on an analytical balance as described by Oelmeier *et al.*, 2010 [57]. A variation coefficient of less than 1.6% was determined for volumes between 20 and 1,000 μL. The concentrations of luminol, HRP, AOX and ethanol were varied in DoE to search for the maximum of luminescence intensity (slope). For quantification of ethanol in samples different dilutions of ethanol (0 to 2 mM) were added to the assay mixture. Finally a 1:8330 dilution of AOX (10–40 U/mg, Sigma-Aldrich) containing of 2 μg of HRP (150 U/mg, Sigma-Aldrich) were added to the assay mixture, containing 0.5 mM luminol (Sigma-Aldrich) dissolved in 5% (v/v) DMSO (Carl Roth) and 95% 40 mM sodium phosphate buffer, pH 8.0. For statistical evaluation, 2.0 mM ethanol was used as a positive control without hydrolase and ester substrate (see also subsection statistical analysis).

All ingredients, except HRP, were mixed at room temperature and then incubated under shaking at 150 rpm at 30°C for 15 min. The reaction was started by adding 15 μL of HRP solution (total activity 0.3 U) to the reaction mixture and mixing with a 96-tip automatic pipette of the evo workstation. Afterwards the 96-well plates were placed in the Infinite M200 Pro reader to measure luminescence. For each measure point luminescence intensity was integrated over 350 ms. Each well was repeatedly measured for up to 1 h. The working temperature was 30°C +/- 2°C. The luminescence raw data was processed either as mean or as numeric integral over the duration of the experiment.

### Oxidative photometric assay

To determine the ethanol concentration the commercial ethanol kit from R-Biopharm AG (Germany) was used. According to the manufacturer, this assay is carried out in a total volume of 3 mL measured in cuvettes at 340 nm in an ordinary spectrophotometer. The manufacturer’s instructions were adapted to the 96-well plate format using 100 μL per well maintaining the ratio of compounds. The assay was started by addition of a 1:10 dilution of aldehyde dehydrogenase solution. The plate was briefly centrifuged to eliminate bubbles. The ethanol concentration was determined by an ethanol calibration curve in the range from 0 to 3 mM (S3 Fig). According to the other assays a positive control for the evaluation test contained only 2.0 mM ethanol without ester and hydrolase (see also subsection statistical analysis).

### pH indicator assay for endpoint measurements

The protocol from Moris-Varas *et al.* was adapted [38] for endpoint measurements. A weak 2.5 sodium phosphate buffer (pK_*a*2_ = 7.2) was adjusted to pH 7.0. Bromothymol blue (pK_*a*_ = 7.1) [58, 59] was dissolved under heating and stirring in this buffer to yield a 0.54 mM stock solution. The assay system contained 10% (v/v) indicator stock solution. According to the other assays a positive control for the evaluation contained only 2.0 mM HCl without ester and hydrolase (see also subsection statistical analysis). Absorbance was measured at the absorption maxima of bromothymol blue at 440 nm and 620 nm in transparent 96-well plates in a conventional plate reader (Epoch, Biotek).

### pH indicator assay for enzymatic activity

The hydrolytic activity was also determined based on *β*-keto acid formation as an indicator of product formation using the pH indicator bromothymol blue. For the determination of activity of hydrolases substrate concentration was 2.0 mM. The calibrations were done by adding different concentrations of HCl (0 to 2 mM) to the buffer in the presence of each tested ester to determine a calibration line (S3 Fig) [39]. During the experiment the pH range was between pH 6.0 to pH 7.0. In this range, the 3-oxo-3-phenylpropanoic acid (*β*-keto acid) is almost completely dissociated. The calculated pK_*a*_for 3-oxo-3-phenylpropanoic acid is 3.56 [60]. For comparison of hydrolases equal masses of protein from 10 mg/mL stock solutions were used. The enzyme concentration of the stock solution was tested by Bradford assay. For each enzyme the blank was determined in buffer with bromothymol blue and without substrate. The incubation temperature was 30°C and 2 mM (concentration *in situ*) ethyl benzoyl acetate was added to the reaction mixture containing hyrolase (see Table 2). The specific activity [*mol*/(*min* · *mg*)] was calculated by the slope at the beginning of the reaction at 620 nm. The specific activity was converted by the number of active sites into the turnover number [1/s].

**Table 2:** Enzymes for screening. For comparison of enzyme activity equal protein concentrations (mg/mL) were used. The concentrations of all hydrolase solutions were determined by Bradford assay. Stock solutions of hydrolases were 10 mg/mL. All enzymes but pNB-Est13 were were purchased comerially. pNB-Est13 was hetrologous expressed in *E. coli*.

**Abbreviation**	**Hydrolase type**	**Origin**	**Molecular weigth**
PPL	Lipase	*Porcine pancreas*	50 kDa [61]
TLL	Lipase	*Thermomyces lanuginosus*	30 kDa [62]
RML	Lipase	*Rhizomucor miehei*	29 kDa [63]
CRL	Lipase	*Candida rugosa*	60 kDa [64]
ALBC	Amano lipase PS	*Burkholderia cepacia*	28 kDa [65]
ALPF	Amano lipase	*Pseudomonas fluorescens*	32 kDa [66]
ALM	Amano lipase M	*Mucor javanicus*	21 kDa [67]
pNB-Est13*	Esterase M	*Bacillus licheniformis*	55 kDa [68]
HRP	Peroxidase	*Armoracia rusticana*	
AOX	Alcohol oxidase	*Pichia pastoris*	

### Statistical analysis

To evaluate the quality of all proposed assays, we used the equations shown in Table 1. For the pH-assay and spectroscopic ethanol-assay μ_*pos*_was the mean absorbance of the positive controls and μ_*neg*_ that of the negative controls. In case of the oxidative luminescence assay μ_*pos*_ was the mean intensity or integral of the positive controls and μ_*neg*_ of the negative controls. For the pH indicator assay the positive control contained 2 mM HCl in 5 mM sodium phosphate buffer (pH 7.2), and the negative control was made from plain buffer. For the two different ethanol based assays, the positive control consisted of assay buffer and 2 mM ethanol, while plain assay buffer was used for the negative controls. Between 44 and 48 positives and negatives were measured for each assay. The measurements took place in 96-well plates. SSMD, student *t*-statistic as well as the KS-statistic and other metrics were computed in R [69]For the *t*-statistic we used the t.test function with Welch correction and for the KS test the ks.test function in R. All other metrics were implemented in R. All plots were created using the ggplot2 library [70]. For *t*-statistics the Welch correction has been designed to account for unequal variances of both groups (positive and negative) [71]. The criteria for evaluation of the assays based on the statistic measures are listed in Table 3.

**Table 3:** Criteria for performance evaluation of HTS-assays [43, 47, 51, 71, 86, 87].

**Z’-factor**	***t*-statistic**	**SSMD**	**Performance**
0.8 ≤ *Z*’ ≤ 1.0	Null-hypothesis rejected	SSMD ≥ 3.0	excellent assay
0.5 ≤ *Z*’ ≤ 0.8			good assay
0.5 ≤ *Z*’ ≤ 0.0			weak assay
0.0 ≤ *Z*’	Null-hypothesis accepted	SSMD ≤ 3.0	“yes/no” type assay

### Software contribution

R is an environment for interactive analysis of statistical data in bioinformatics offering many additional software packages. We combined all approaches and implemented the AssayToolbox package in R to make the applied methods accessible to a wide community. Software link: http://www.cbs.tu-darmstadt.de/htsassay.zip

## Results

**Fig 1:**
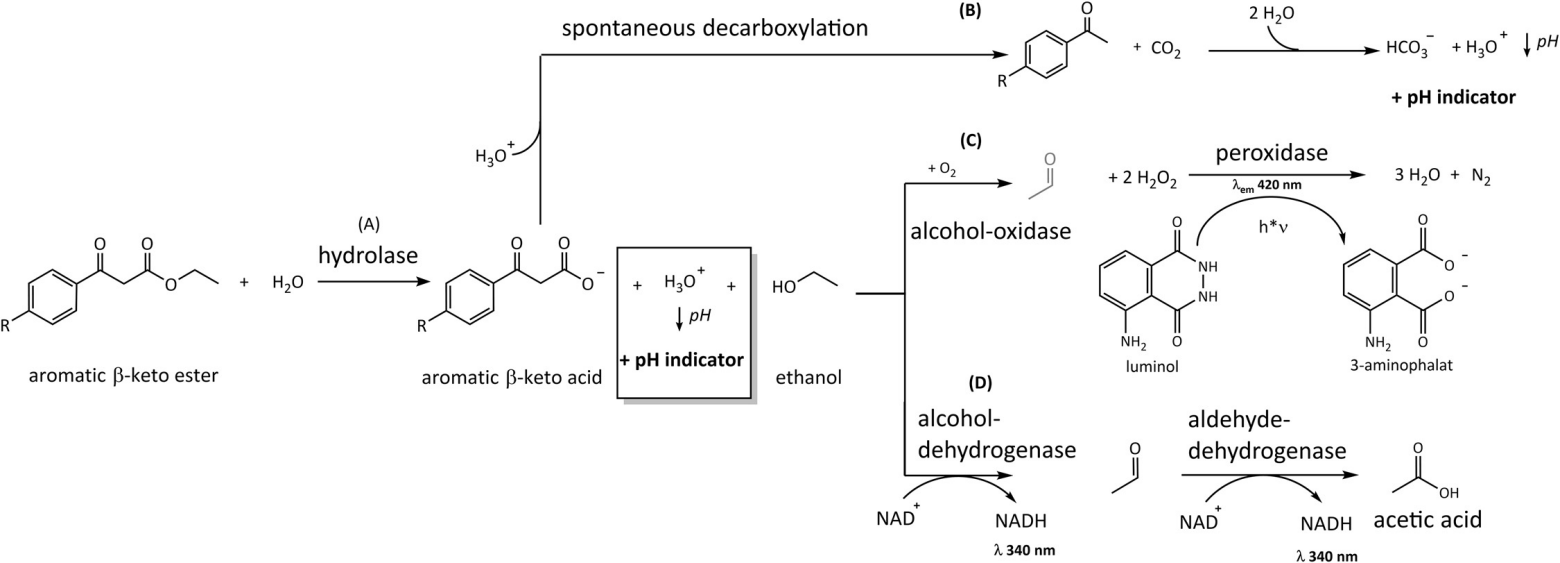
Overview of assays for hydrolysis of *β*-keto esters. (A) Hydrolysis reaction of *β*-keto ethylester catalyzed by hydrolase. (B) pH-assay: photometric detection of pH change due to acid formation and deprotonation with bromothymol blue. Ethanol based assays: (C) Oxidative luminescence assay using alcohol oxidase, horseradish peroxidase and ethanol (D) Photometric detection of ethanol by oxidation by dehydrogenases under conversion of NAD^+^to NADH.

Upon hydrolysis of *β*-keto ethylesters, ethanol is released and a *β*-keto acid is formed. Afterwards the acids can decarboxylate to carbon dioxide and acetophenone. A small amount of carbon dioxide forms carbonic acid in water, which lowers the pH-value as well [72] (Fig 1). We used the pH shift (Fig 1(B)) visualized by the indicator bromothymol blue as a measure of product formation. We choose this indicator due to its expected pH value of the enzyme coupled system consisting of lipase and transaminase. In addition we created a new luminometric assay (Fig 1(C)) for use in 96-well plates. This assays detects the formation of the product ethanol by an enzymatic reaction. Ethanol was oxidized by alcohol-oxidase to yield acetaldehyde and hydrogen peroxide, which is used to oxidize luminol to 3-aminophthalate by horseradish peroxidase (Fig 1(C)). This well-known chemiluminescent reaction is frequently used in immunoassays such as Western Blot or ELISA [73]. For the luminescence-based assay we expected a higher sensitivity as the emitted light can be accumulated (high signal) and there is no stray light from excitation (low background) [74]. Other alcohols can also be oxidized by alcohol oxidase [75] so that this assay would be applicable to the hydrolysis of different types of esters. For comparison we used the commercial ethanol assay from r-Biopharm, based on the oxidation of ethanol to acetic acid in a two-step oxidation by alcohol dehydrogenase and aldehyde dehydrogenase [40]. In this reaction cascade, two equivalents of NADH are generated, which can be detected by UV/Vis spectroscopy at 340 nm. The aim was to identify the best assay out of these three, i.e. the one with the highest dynamic range and most stable readout. The system also has to be sensitive enough to operate reliably with small quantities of substrate and enzymes to save costs in high-throughput screening.

The first step was to optimize all assays by testing different enzyme and compound concentrations without hydrolases, with HCl or ethanol as signal inducing molecules, mostly for the luminescence assay. For comparison of the three HTS assays, endpoint measurements were done. The two step oxidative luminescence assay was optimized by design of experiments (DoE) to maximize the duration of luminescence and the sensitivity for endpoint measurements. The isochronic induction of luminescence in all wells was very important for the reliable quantification of ethanol. The assay mixtures were automatically pipetted by the Tecan evo pipetting robot that can simultaneously pipette 96 wells. It turned out that a pre-incubation time of 15 min without peroxidase was necessary. Without pre-incubation, the resulting luminescence signal was too weak for the quantification of ethanol. Luminescence enhancers like 4-iodophenol [76], were added in order to maximize the luminescence output, however no sufficient signal amplification was observed so that these additives were omitted. Due to the relatively weak signal the luminescence assay was only suitable for endpoint determinations. For quantification, we calculated both the numeric integral and the mean of luminescence over the measured time. A clear luminescence signal was detectable for about 50 min.

Likewise, the commercial spectrometric ethanol assay was only suitable for endpoint determinations when carried out in 96-well plates. The assay was tested for kinetic measurements with the result that no correlation between substrate concentration and reaction rate of hydrolase was observed. The pre-assembled commercial assay might not be suitable for kinetics, because the concentration of NAD and alcohol-dehydrogenase can not separately be varied. In contrast to these two assays, the pH indicator assay has no additional enzymes. The pH indicator, bromothymol blue, has two absorption maxima in the UV-Vis spectrum at 440 and 620 nm and a pK_*a*_at the desired pH value for the cascade reaction of a hydrolase in combination with a *ω*-transaminase. The extinction coefficient at both wavelengths depends on the pH-value so that both were used for evaluation measurements.

**Fig 2:**
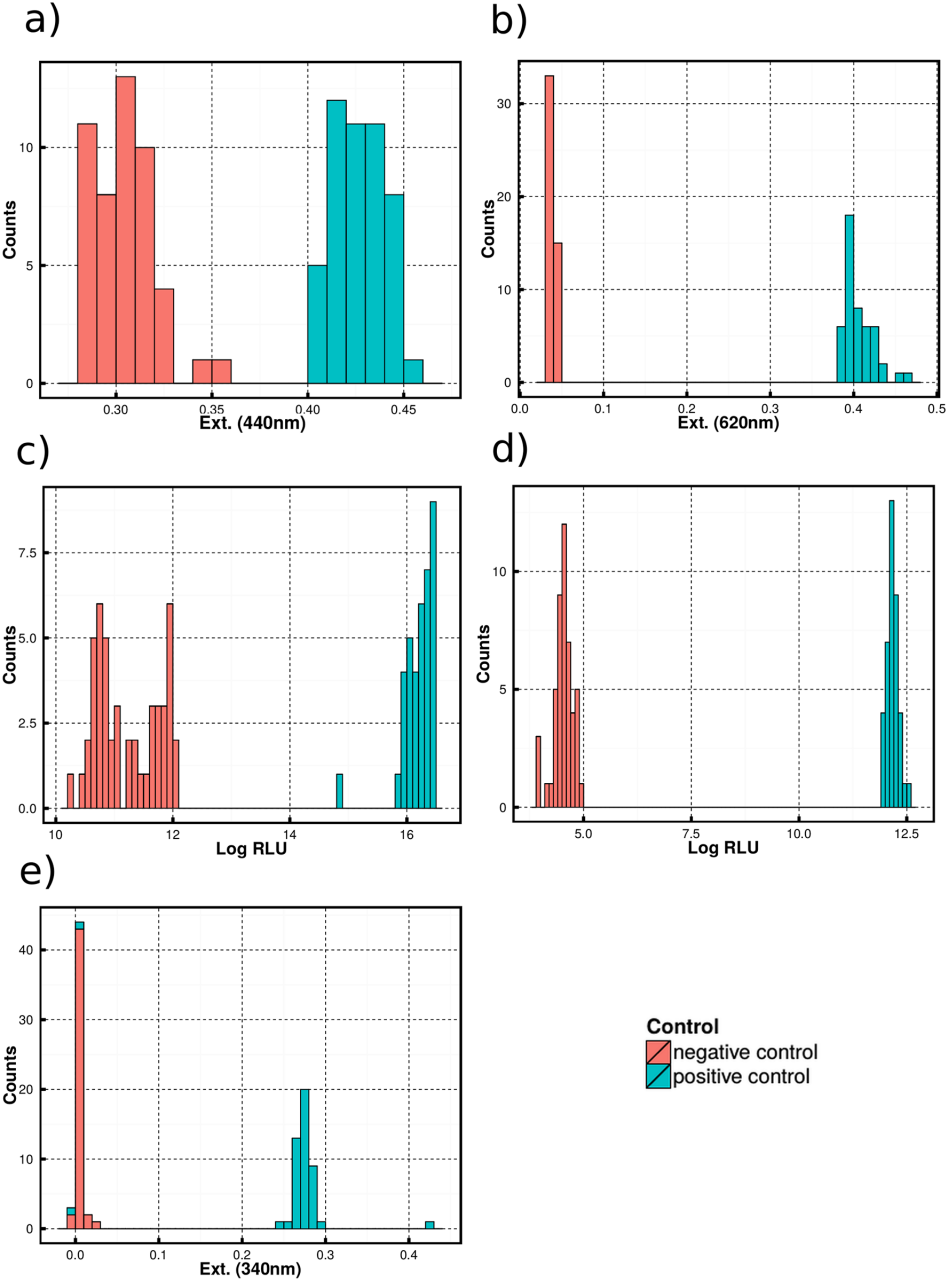
Histogram of positive and negative controls of different HTS assays. For each control 44–48 values were measured a) pH-indicator assay at 440 nm b) pH-indicator assay at 620 nm c) luminescence ethanol assay (mean luminescence intensity) d) luminescence ethanol assay (integrated luminescence intensity) e) photometric ethanol assay at 340 nm.

To evaluate these assays, we performed simple tests with positive and negative controls without the real substrate and hydrolases. One-half of each plate was filled with the positive control and the other half with the negative control. For the pH-assay, we used 2 mM HCl as positive control, which corresponds to the maximum product concentration, if all substrate was converted. For evaluation of the oxidative luminescence and spectrometric ethanol assay, we added 2.0 mM ethanol as positive control for the tested enzyme coupled assays. Reaction buffer without substrate and hydrolase was used as the negative control for all assays. In Fig 2 the distribution of negative and positive controls for each assay is shown. For evaluation we used the criteria for evaluation listed in Table 3.

The KS-statistic value was almost equal for all assays, because all distributions seem to have a maximal distance. KS-statistic shows that all positives and negatives have a clearly separated distribution, but it cannot answer the question whether the degree of separation is acceptable for HTS. Consequently KS-statistic test is not necessarily helpful to compare the performance of these types of assays. Z’-factor, *t*-statistic and SSMD allowed for a more detailed evaluation: Due to the low performance in all statistical tests the Lum(int) and pH-based assay at 440 nm were considered unsuitable and were rejected. The pH-based assay at 620 nm performed best, because it has the largest dynamic range (Fig 2(b)) and thus is a reliable test for the applicability of our statistical measures.

**Fig 3:**
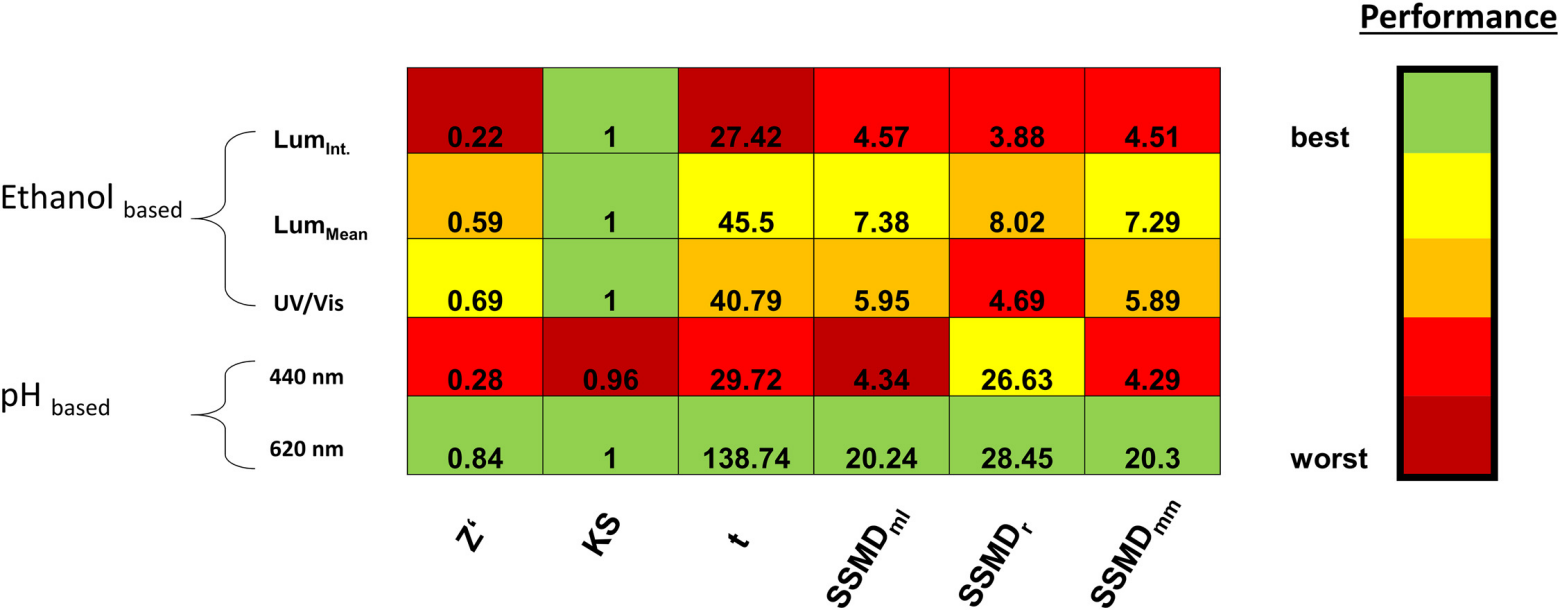
Matrix of different statistical parameters for evaluation of HTS assays. For each parameter the assays were ranked from the best (green) to the worst assay (red). The assays were grouped by the kind of detection. Ethanol quantification: Lum(int), Lum(mean), UV/Vis (ethanol dehydrogenase assay). pH-indicator assay at 440 and at 620 nm.

SSMD yielded similar results as the Z’-factor and student *t*-test, showing that this measure, that was developed for RNAi screens, can be employed for the evaluation of enzymatic reactions (Fig 3). Robust SSMD_*R*_ differs from all metrics for the pH-based assay at 440 nm which indicates that outliers exist for this assay. The negative consequence of outliers can be avoided, if at least triplicates are measured. In contrast to all other measures SSMD_*R*_ is suitable for selection of sensitive assays with more outliers. On the one hand, additional metrics like the mentioned SSMD_*R*_ are particular useful in case of assays with outliers, because in some screening approaches they have a minor influence on the evaluation. However on the other hand statistics depend on explanatory power of the experimental data. As a conclusion of this, it should be carefully considered which metrics as well as processed raw data are suitable for a certain HTS evaluation.

**Fig 4:**
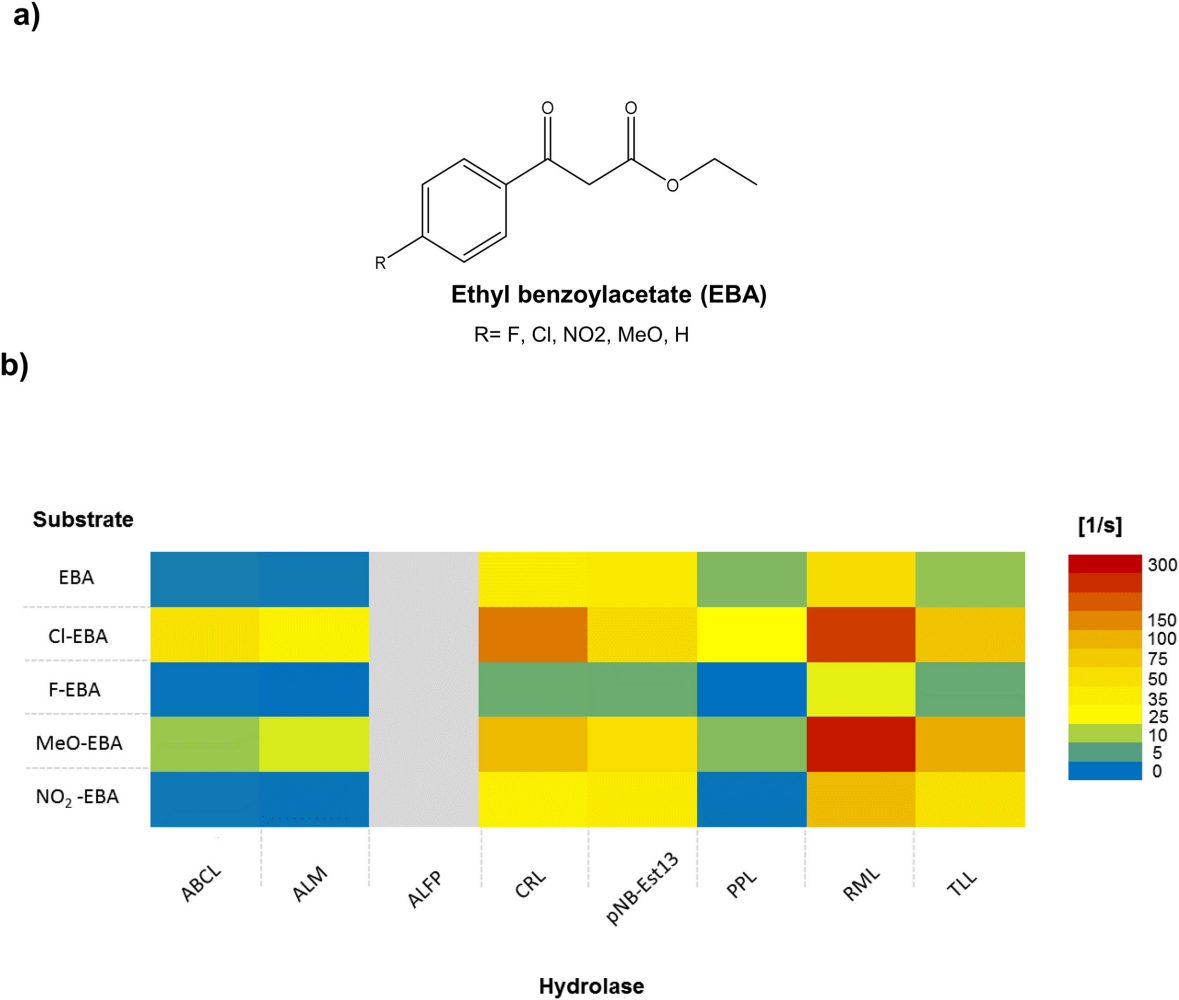
Screening of different *β*-keto esters against different hydrolases with pH-indicator assay. (for abbreviations see Table 3) The concentration of substrates was 2.0 mM in 2.5 mM sodium phosphate buffer (pH 7.0). The reactions were carried out at 30°C for 30 min. The activities (μmol/min) were normalized to μmol actives sites per s. (grey: no measurement possible) Para-nitrobenzyl-esterase 13 (pNB-Est13) was purified by us (described in methods). a) Structure of ethyl benzoylacetate with different substituents used as substrates in the screening. b) Activity matrix for 8 different enzymes (ABCL, ALM, ALFP, CRL, pNB-Est13, PPL, RML and TLL) against the respective substrates. The turnover number is in [1/s], for illustration turnover values were color coded from blue (low) to red (high).

As the pH indicator assay at 620 nm obtained the highest statistical scores it was chosen for a subsequent small scale screening of a set of different hydrolases with different derivates of ethyl benzoyl acetate (EBA; Fig 4). First of all, we determined for each ester a calibration curve with HCl concentrations between 0 and 2 mM at 620 nm (S3 Fig). The strong acid HCl (pK_*a*_ = -6) nearly dissociates to 100% in a wide range of the pH-scale. In contrast the weak *β*-keto acid (pK_*a*_ = 3.56) can only completely dissociate, when the pH is clearly above the pK_*a*_. A weak sodium phosphate buffer (pH 7.0) was very important to of the pH change during the reaction. The range of pH-values during the experiment was between pH 6 and pH 7. This was the prerequisite for the calculation of the enzyme activity at the beginning of an activity test by the absorbance over the time. The concentrations were calculated by the different calibration curves of each substrate.

We tested each hydrolase in combination with each ester to determine which hydrolases are the most active ones and which substrates have the highest accessibility (Fig 4).

When adding the enzymes to the reaction buffer containing bromothymol blue we observed a color change for Amano lipase. However, upon substrate addition we detected no change in pH-value, so we assume that interactions between the enzyme and the pH-indicator, a polyaromatic molecule, may have interfered with the reaction or its colorimetric detection. Therefore, it was not possible to screen the activity of Amano lipase of *Pseudomonas fluorescens*(ALFP). Such interfering effects of indicator molecules and enzymes are well known, see for instance Banyai [12, 77]. An alternative option in pH-indicator screening might be the use of 4-nitrophenol (pK_*a*_ = 7.16) as an indicator molecule. It has only one aromatic ring and is therefore less hydrophobic [77, 78]. None of the other hydrolases showed any change in absorbance with only the pH indicator in the absence of substrate.

The experiment revealed that every substrate was converted by hydrolases. The lipase from *Rhizomucor miehei*(RML) showed the highest activity of the tested enzymes while the porcine pancreas lipase mix (PPL) showed the lowest hydrolysis activity towards aromatic *β*-keto esters. The only esterase in the screening (pNB-Est13), showed the fourth highest activity for all substrates. This shows that esterases may be considered as alternatives to lipases. This is of interest as the activity of lipases depends on hydrophobic surface interactions so that the substrate spectrum is limited. Esterases could help to broaden the substrate range for enzymatic *β*-keto acid and *β*-amino acid synthesis towards more hydrophilic compounds. Taken together, the assay results confirm, that the pH-assay at 620 nm can indeed be used for substrate-hydrolase screenings, albeit other indicator dyes may have to be tested to reduce non-specific interactions.

To explain the high activity of RML against all substrates, we consider the previous work of Rehm *et al.* [79]. Thus we compared the active site volumes and structural conformations like the opened as well as closed state of RML,*Candida rugosa* lipase (CRL) and TLL [79]. For example, in water, the lipase active site is covered by a mobile element, the lid, which opens upon substrate binding due to the hydrophobic interface [63]. In contrast to RML and TLL, CRL possesses a large and complex lid (residues 66–92), consisting of a short and a long *α*-helix. A comparison of the closed and open crystal structure of CRL revealed that the lid has to refold partially (S1b Fig). As expected for this reason, a slower opening and closing when compared to RML and TLL could be shown using Molecular Dynamics [79]. In contrast to CRL, a fast rigid body movement of the lid was suggested for RML and TLL. This opening event is suggested to be the rate limiting step during catalysis [64]. In addition several studies proposed that conformational rearrangement during the catalysis could have a much greater influence on the activity than binding energy inside the substrate pocket [80–82]. The discussion on the influence of the dynamic on the activity of the enzyme is still on going [83]. Although CRL possesses a even bigger lid than RML and TLL, the same approximated active site volume was calculated for all three lipases (RML: 59.5 nm^3^; CRL 60.3 nm^3^; TLL 57.3 nm^3^, S1 Fig)

Beside this, the substrate properties of the ethyl benzoyl acetate substituents might have an influence on the activity caused by polarization and steric differences.

## Conclusion

We compared three different assays based on a set of positive and negative controls by end point measurements. For this purpose we applied for the first time an alcohol oxidase-peroxidase-luminescence assay for ethanol quantification in 96-well format. Additionally, we evaluated a pH-indicator and a commercial ethanol assay for HTS. We applied several statistical measures for biocatalysis assay evaluation and found that classical Z’-factor, SSMD and *t*-statistic can indicate whether an assay is suitable for HTS. The pH-indicator assay based on color change of bromothymol blue at 620 nm upon acid formation performed best. However, strictly considered is the most accurate way to evaluate HTS assays to test the complete coupled assays (consisting of: enzyme(s) of interest, substrate(s) and assay compounds), then all necessary assay compounds, substrates and catalysts have to be tested in combination with each other. Otherwise possible cross-interactions of all assay substances can not be excluded when not the complete system is tested. But this approach is not suitable for a screening with the variation of more than one compound, caused by the exponentially growing number of experiments when two or more different compounds are tested. A further obstacle for evaluation of HTS assays by the whole testing system is that the hydrolases have to be previously inactivated in presence of the substrate for an end-point measurement. Moreover, the limiting factors for an evaluation are often enzymes as well as substrates availability (e.g. environmental samples). Therefore, a robust reduced evaluation approach might be suitable and weighed against potential benefits of a complete coupled system. In addition the non-coupled evaluation system could be also adapted for more complex screenings e.g. in case for enzymes from microbial lysates. To accomplish this a standard lysate from the expression host could be included into the evaluation setup. Beside this, using the pH assay in a screen of a panel of mostly commercially available lipases we identified the RML as the most efficient enzyme for the hydrolysis of the tested set of aromatic *β*-keto ethyl esters. We were able to explain those findings by structural models of the enzymes’ binding pocket and lid as well as by comparison of our data with descriptive literature on lipases dynamics, including MD.

Furthermore, we demonstrated that the esterase pNB-Est-13 is also suitable for aromatic *β*-keto ester hydrolysis. This may help to broaden the substrate spectrum since esterases may accept more hydrophilic substrates than lipases.

## Outlook

The pH-assay is a very useful method in the search for efficient hydrolases for a cascade reaction as it is robust, inexpensive and allows to record reaction kinetics. Our best-performing hydrolase, RML, could be used in combination with *ω*-transaminase, in cascade reactions for the synthesis of *β*-amino acids. All tested *β*-keto esters could be utilized for cascade reaction with hydrolase and *ω*-transaminase [84]. The newly proposed enzymatic oxidative luminescence assay, still requires further optimization. However, it bears the potential for a more sensitive assay, due to the cumulative measurement and the lower background compared to fluorescence and absorption measurements [85].

Beside this, we want to extend our modelling efforts to validate our hypothesis for the activity of the RML and to make predictions for the activity lipases and esterases like the pNB-Est-13, with a broad substrate tolerance.

## Supporting Information

S1 Fig Structural investigation of RML and CRL. a) Surface representation of CRL open (grey, 1CRL) and closed (blue, 1THG) state. Structural alignment of CRL open (grey, 1CRL) and closed (blue, 1THG). b) Surface representation of RML open (grey, 4TGL) and closed (blue, 3TGL) state. Structural alignment of RML open (grey, 4TGL) and closed (blue, 3TGL). c) Cavity volume (blue) of CRL in profile and front view. d) Cavity volume (blue) of RML in profile and front view.
(TIF)

S2 Fig pNB-Est13 esterase compared by SDS-PAGE. Equal volumes with the same protein concentration were analyzed. The separation was carried in in a 12.5% SDS- polyacrylamide-gel at 200 V. Crude pNB-Est13 esterase was used after osmotic shock with TES-buffer and ddH_2_O.
(TIF)

S3 Fig Calibration of HTS-assays. The error bars show standard deviation. (a) Spectrometric ethanol assay (Pearson: 0.998, p-value = 1.64 ⋅ 10^−8^) (b) pH-indicator assay at 620 nm (Pearson: -0.999, p-value = 2.47 ⋅ 10^−8^). (c) Mean Luminescence ethanol assay (Pearson: 0.967, p-value = 8.15 ⋅ 10^3^) a) and b) were measured in triplicate. The assays are describe in the methods section.
(TIF)

S4 Fig First step optimization of the luminescence assay, by varying the concentration of luminol/ethanol (a) and luminol/hydrogen peroxide (b) against the average luminescence intensity over the time. The surface model plot was created by Modde 10.1.
(TIF)

S5 Fig Contour plot to optimize the luminescence assay. The ethanol concentration (0 to 2.5 mM) was plotted against the HRP concentration (0 to 0.87 U/mL),AOX concentration (0 to 18.5 mU/mL)and luminol concentration (0 μM;37.5 μM;75 μM). Was scaled Contrasting the activity. A) luminescence slope per s: blue = high activity; red = low / no activity. (B) averaged luminescence: red = high activity blue = low / no activity. The plot was created by Modde 10.1.
(TIF)
