# Supplementary figures and images for: Statistical Evaluation of HTS Assays for Enzymatic Hydrolysis of β-Keto Esters
Source: PLoS One. 2016 Jan 5;11(1):e0146104. doi: 10.1371/journal.pone.0146104 (PMC4711668; doi:10.1371/journal.pone.0146104)

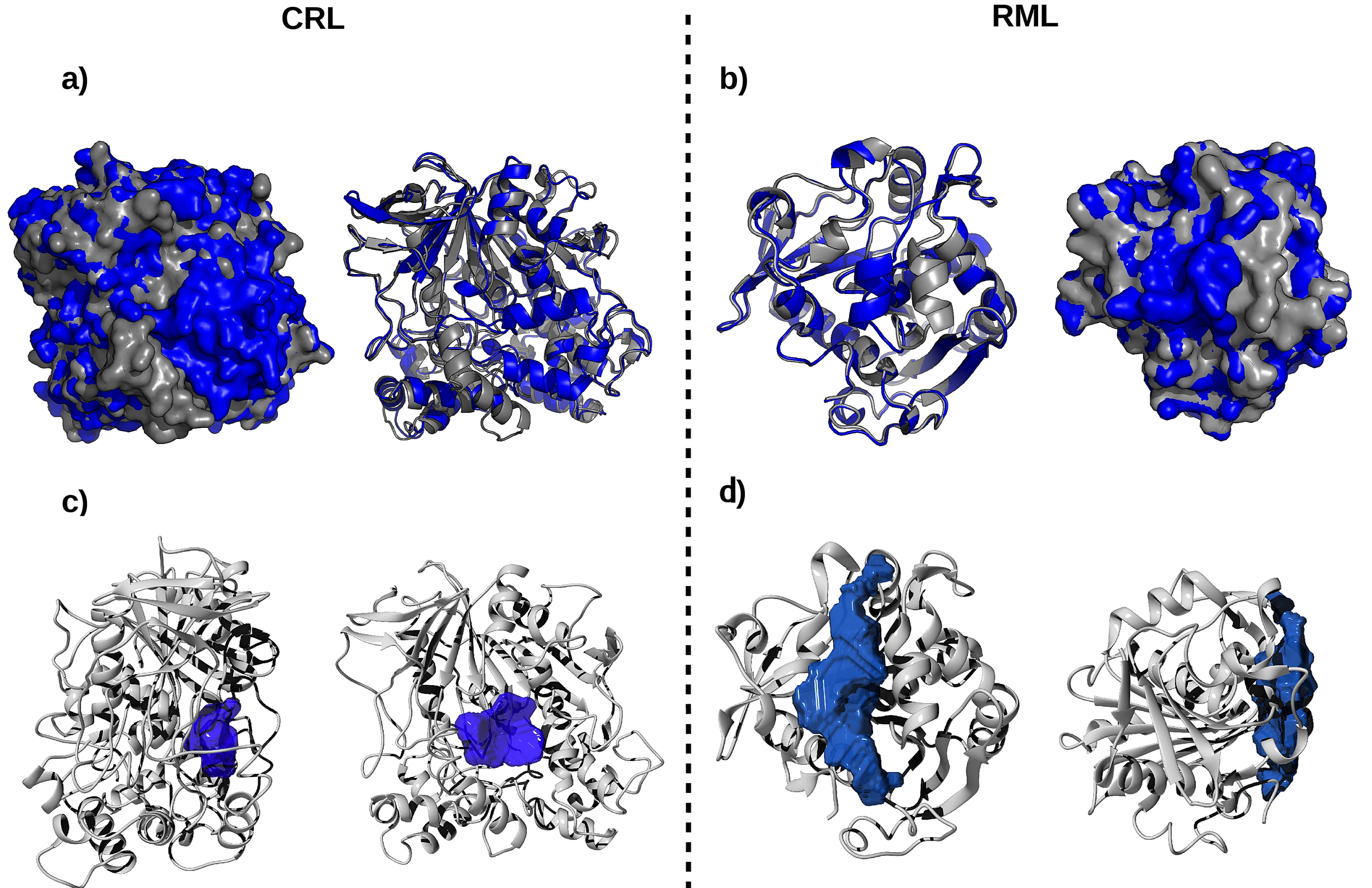

Supplement: S1 Fig — a) Surface representation of CRL open (grey, 1CRL) and closed (blue, 1THG) state. Structural alignment of CRL open (grey, 1CRL) and closed (blue, 1THG). b) Surface representation of RML open (grey, 4TGL) and closed (blue, 3TGL) state. Structural alignment of RML open (grey, 4TGL) and closed (blue, 3TGL). c) Cavity volume (blue) of CRL in profile and front view. d) Cavity volume (blue) of RML in profile and front view. (TIF) [file pone.0146104.s001.tif]

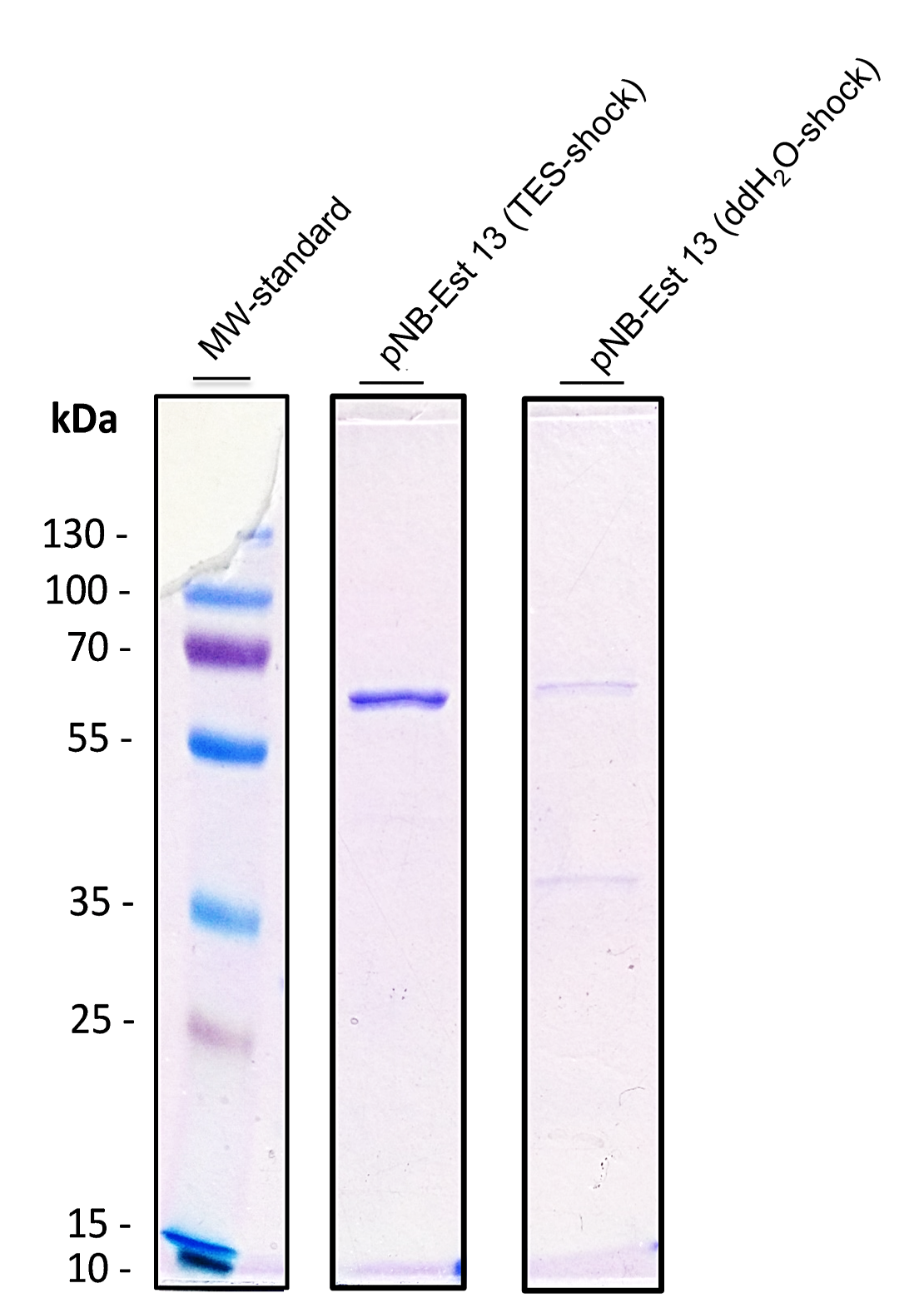

Supplement: S2 Fig — Equal volumes with the same protein concentration were analyzed. The separation was carried in in a 12.5% SDS- polyacrylamide-gel at 200 V. Crude pNB-Est13 esterase was used after osmotic shock with TES-buffer and ddH2O. (TIF) [file pone.0146104.s002.tif]

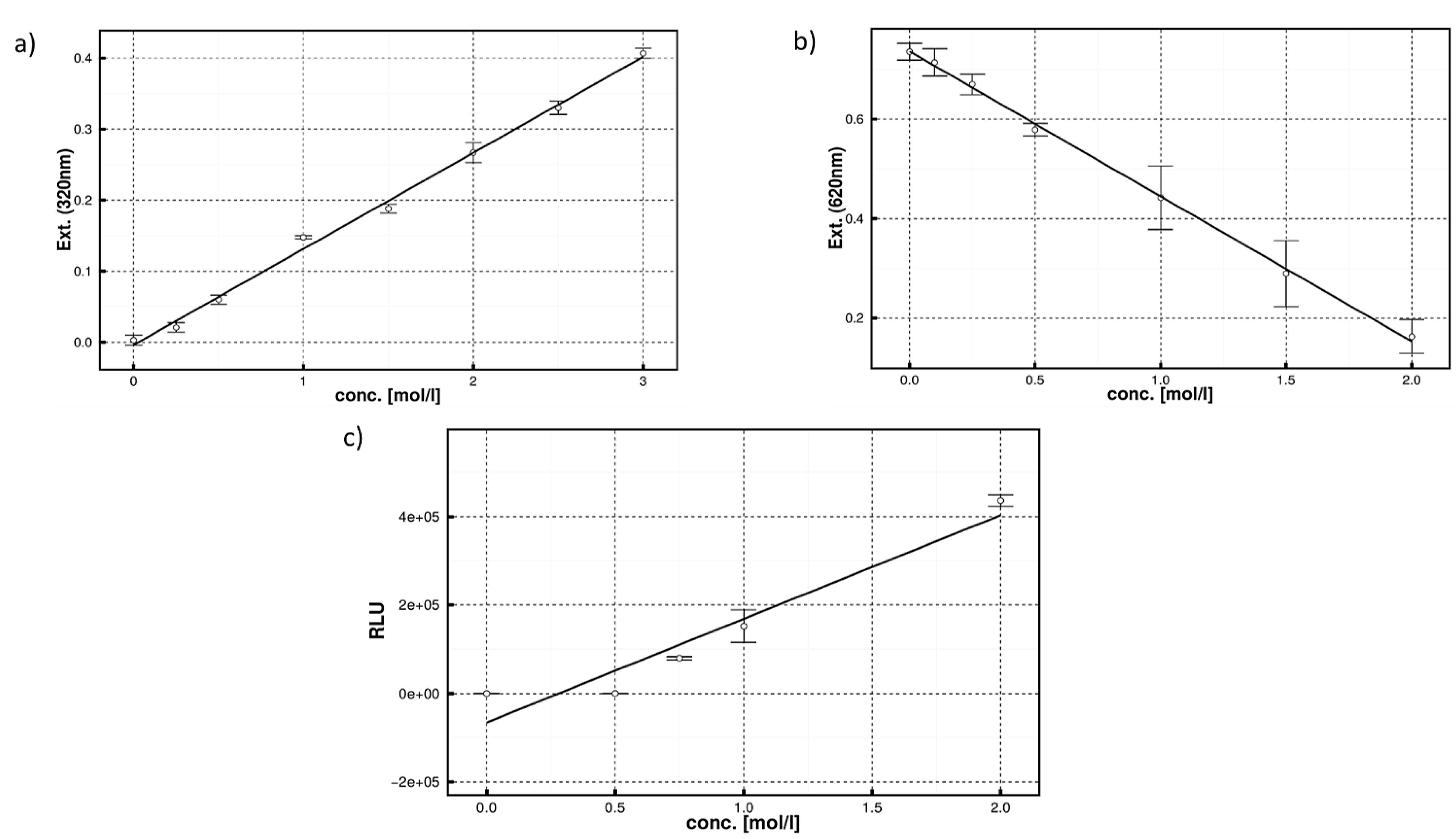

Supplement: S3 Fig — The error bars show standard deviation. (a) Spectrometric ethanol assay (Pearson: 0.998, p-value = 1.64 ⋅ 10−8) (b) pH-indicator assay at 620 nm (Pearson: -0.999, p-value = 2.47 ⋅ 10−8). (c) Mean Luminescence ethanol assay (Pearson: 0.967, p-value = 8.15 ⋅ 103) a) and b) were measured in triplicate. The assays are describe in the methods section. (TIF) [file pone.0146104.s003.tif]

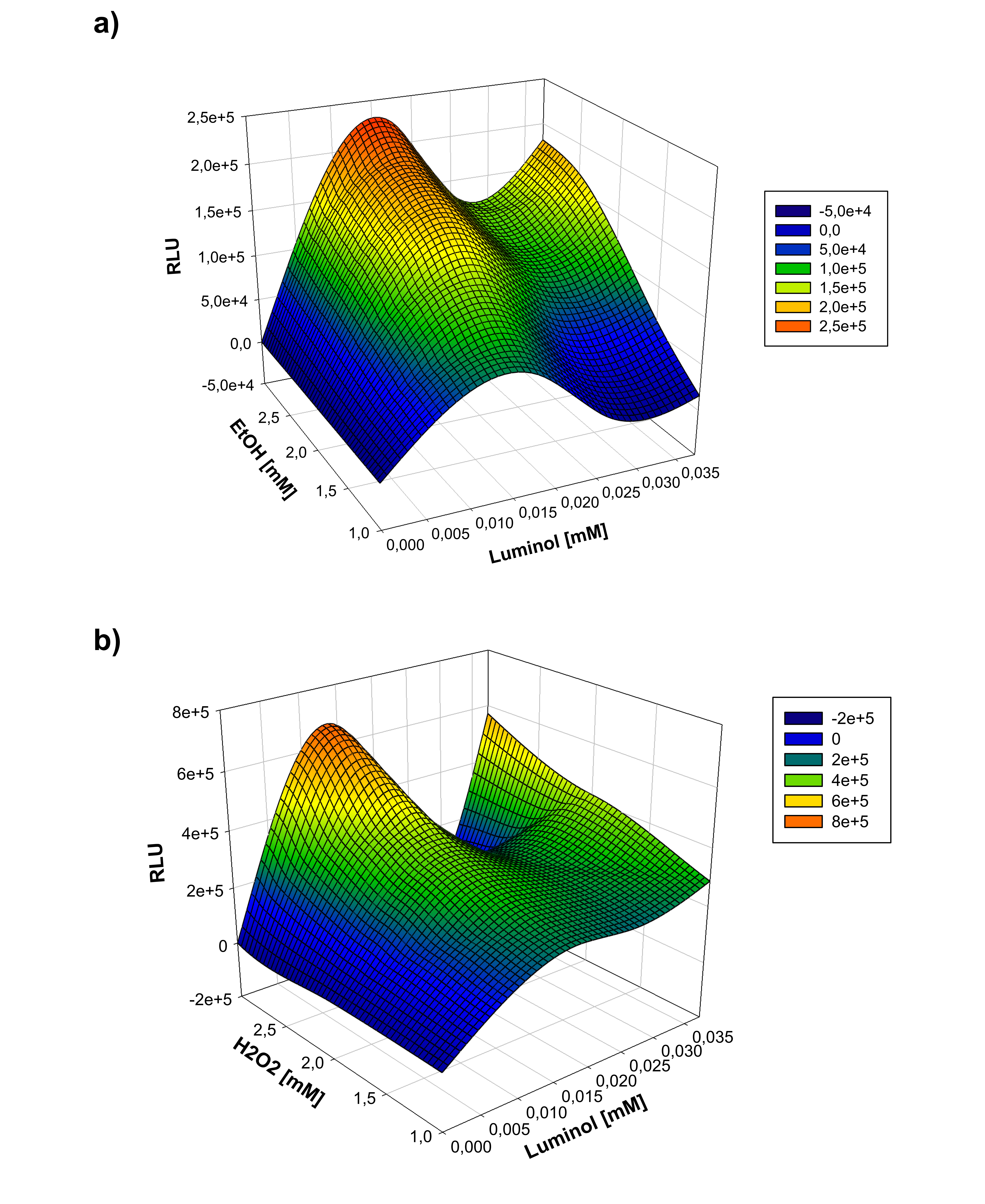

Supplement: S4 Fig — The surface model plot was created by Modde 10.1. (TIF) [file pone.0146104.s004.tif]
